# Glaucoma Patients Have a Lower Abundance of Butyrate-Producing Taxa in the Gut

**DOI:** 10.1167/iovs.65.2.7

**Published:** 2024-02-05

**Authors:** Joëlle E. Vergroesen, Zakariya A. Jarrar, Stefan Weiss, Fabian Frost, Abdus S. Ansari, Picard Nguyen, Robert Kraaij, Carolina Medina-Gomez, Henry Völzke, Frank Tost, Najaf Amin, Cornelia M. van Duijn, Caroline C. W. Klaver, Clemens Jürgens, Chris J. Hammond, Wishal D. Ramdas

**Affiliations:** 1 Department of Ophthalmology, Erasmus MC University Medical Center, Rotterdam, The Netherlands; 2 Department of Epidemiology, Erasmus MC University Medical Center, Rotterdam, The Netherlands; 3 Department of Ophthalmology, King's College London, London, United Kingdom; 4 Department of Twins Research and Genetic Epidemiology, King's College London, London, United Kingdom; 5 Interfaculty Institute of Genetics and Functional Genomics, University Medicine Greifswald, Greifswald, Germany; 6 Department of Medicine A, University Medicine Greifswald, Greifswald, Germany; 7 Department of Internal Medicine, Erasmus MC University Medical Center, Rotterdam, The Netherlands; 8 Institute for Community Medicine, University Medicine Greifswald, Greifswald, Germany; 9 Department of Ophthalmology, University Medicine Greifswald, Greifswald, Germany; 10 Nuffield Department of Population Health, University of Oxford, Oxford, United Kingdom; 11 Department of Ophthalmology, Radboud University Medical Center, Nijmegen, The Netherlands; 12 Institute of Molecular and Clinical Ophthalmology, University of Basel, Basel, Switzerland

**Keywords:** glaucoma, intraocular pressure (IOP), gut, microbiome, butyrate

## Abstract

**Purpose:**

Glaucoma is an eye disease that is the most common cause of irreversible blindness worldwide. It has been suggested that gut microbiota can produce reactive oxygen species and pro-inflammatory cytokines that may travel from the gastric mucosa to distal sites, for example, the optic nerve head or trabecular meshwork. There is evidence for a gut-eye axis, as microbial dysbiosis has been associated with retinal diseases. We investigated the microbial composition in patients with glaucoma and healthy controls. Moreover, we analyzed the association of the gut microbiome with intraocular pressure (IOP; risk factor of glaucoma) and vertical cup-to-disc ratio (VCDR; quantifying glaucoma severity).

**Methods:**

The discovery analyses included participants of the Rotterdam Study and the Erasmus Glaucoma Cohort. A total of 225 patients with glaucoma and 1247 age- and sex-matched participants without glaucoma were included in our analyses. Stool samples were used to generate 16S rRNA gene profiles. We assessed associations with 233 genera and species. We used data from the TwinsUK and the Study of Health in Pomerania (SHIP) to replicate our findings.

**Results:**

Several butyrate-producing taxa (e.g. *Butyrivibrio*, *Caproiciproducens*, *Clostridium sensu stricto 1*, *Coprococcus 1*, *Ruminococcaceae UCG 007*, and *Shuttleworthia*) were less abundant in people with glaucoma compared to healthy controls. The same taxa were also associated with lower IOP and smaller VCDR. The replication analyses confirmed the findings from the discovery analyses.

**Conclusions:**

Large human studies exploring the link between the gut microbiome and glaucoma are lacking. Our results suggest that microbial dysbiosis plays a role in the pathophysiology of glaucoma.

Glaucoma is an eye disease that is the most common cause of irreversible blindness worldwide.^1^ More than 80 million people have glaucoma globally and this estimate is predicted to double by 2040.^2^ Glaucomatous visual field loss typically starts in the periphery and can progress to involve the central visual field, resulting in an irreversible decline in visual acuity. Intraocular pressure (IOP) is currently the only modifiable risk factor to target the progressive loss of retinal ganglion cells (RGCs) in glaucoma. However, IOP-independent mechanisms may play a role in its etiology given that some people develop glaucoma without an elevated IOP^3^ and, in others, the visual field deterioration progresses despite an apparently sufficient reduction in IOP.^4^ Furthermore, there are people with ocular hypertension who do not convert to glaucoma.^5^ Clinical signs of glaucomatous damage include excavation of the optic nerve head, quantified as the vertical cup-to-disc ratio (VCDR).^6^

Several studies have shown that obesity is associated with a higher risk of developing glaucoma and an elevated IOP.^7^^–^^12^ On the other hand, microbiome dysbiosis has been widely acknowledged to play a role in the etiology of obesity.^13^^,^^14^ Dysbiosis of the gut microbiome has been linked to low-grade inflammation in people with obesity.^15^^,^^16^ In addition, there is accumulating evidence suggesting neuroinflammation to be a crucial component in glaucoma.^17^^,^^18^ Therefore, the link between the gut microbiome and glaucoma has gained increased attention. A recent study provided compelling evidence that the CD4^+^ T-cells are involved in the pathogenesis of chronic neurodegeneration in the eye of mice after pre-sensitization by commensal microflora.^19^ A few studies, based on oral microbiota and not gut, linked the microbiome to glaucoma.^20^^–^^22^ Potentially, gut microbiota can produce reactive oxygen species and pro-inflammatory cytokines that may travel from the gastric mucosa to distal sites, such as the optic nerve head or trabecular meshwork.^23^ Furthermore, the gut microbiota may also influence the production and availability of neuroprotective factors that could in turn promote RGC survival.^24^

Although these recent findings are encouraging, to date, there are few small human studies exploring the link between the gut microbiome and glaucoma. The functional differences between gut microbiome composition of participants with and without glaucoma may help increase our knowledge of the role and impact of the microbiome in glaucoma. Therefore, using multiple cohorts, we analyzed the microbial composition in patients with glaucoma and healthy controls. Furthermore, we assessed its association with IOP and VCDR.

## Methods

### Discovery Cohort and Analyses

#### Study Populations and Available Data

The RS^25^ is approved by the Medical Ethics Committee of the Erasmus University Medical Center (Erasmus MC; registration number MEC 02.1015) and by the Dutch Ministry of Health, Welfare, and Sport (Population Screening Act WBO, license number 1071272-159521-PG). All participants provided written informed consent. We used data from the second follow-up of the third RS cohort (RS-III-2). Participants underwent extensive eye examinations, including Goldmann applanation tonometry (Haag-Streit AG, Bern, Switzerland), color fundus photography centered on the macula and on the optic disc (Topcon TRC 50EX [Tokyo Optical Co, Tokyo, Japan] and the Sony DXC-950P [Sony Corporation, Tokyo, Japan] digital camera), optical coherence tomography (OCT) centered on the macula and the optic disc (OCT-2000; Tokyo Optical Co.), and visual field testing (Humphrey Field Analyzer; [HFA] II 740; Carl Zeiss, Oberkochen, Germany). Details on visual field testing have been described previously.^26^ In short, all participants underwent visual field testing. When a visual field defect appeared to be present, a second supra-threshold test was performed. If the second supra-threshold test showed at least one overlapping abnormality in the same hemifield, a full-threshold HFA was performed on both eyes. If abnormalities were consecutive and reproducible, the visual field loss was considered to be present. Defects had to be in a consistent hemifield and at least one depressed test point had to have exactly the same location on all fields. Other possible causes of visual field loss were excluded by examining fundus photographs, ophthalmic examination reports, medical histories, and magnetic resonance imaging (MRI) scans of the brain. Discrepancies were resolved by consensus. Glaucoma cases had an open anterior chamber angle and no history or signs of secondary glaucoma.^26^ For IOP, three measurements were taken from each eye, the median value of which was recorded.^27^ VCDR was calculated as the ratio of the vertical diameter of the cup against the vertical diameter of the optic disc. For glaucoma cases, we used measurements of the affected eye. If both eyes were affected or unaffected, a random eye was selected.

From January 2021 onward, based on their diagnosis treatment combination (DBC)/International Classification of Disease-10th revision (ICD-10) code, patients with glaucoma from outpatient clinics of Dutch hospitals were invited to participate in the present study. The erasmus glaucoma cohort (EGC) is approved by the Medical Ethics Committee of the Erasmus MC (registration number MEC 2020-0872). All participants provided written informed consent. We had extensive eye data available, including glaucoma diagnosis and IOP. All patients were profiled for the gut microbiome and received multiple questionnaires.

#### Fecal Sample Collection and Microbiome Profiling

A detailed description on how the gut microbiome composition was generated is available elsewhere.^28^ In short, all participants were instructed to collect a stool sample at their home in sterile tubes (RS) or tubes from the OMNIgene GUT kit (DNA Genotek, Ottawa, Canada; EGC) and to send the sample by regular mail to the research location of Erasmus MC. Upon arrival, samples were checked and stored at −20°C. For RS, samples were excluded if they were underway for more than 3 days.^28^ For RS, an automated stool DNA isolation kit (Diasorin, Saluggia, Italy) was used to isolate bacterial DNA from approximately 300 mg stool aliquot using a bead-beating step. For EGC, 1 mL of homogenized stool was taken from the Gentotek tube, bead beated, and subjected to further DNA isolation using the InviMag Stool DNA Kit (INVITEK Molecular, Berlin, Germany). The V3 and V4 hypervariable regions of the bacterial 16 S rRNA gene were amplified and sequenced on an Illumina MiSeq platform with the V3 kit (2 × 300 bp paired-end reads; Illumina). Reads were subsampled at 10,000 reads per sample. Raw reads from Illumina MiSeq were demultiplexed using a custom script to separate sample fastq files based on the dual index. Primers, barcodes, and heterogeneity spacers were trimmed off using tagcleaner version 0.16.^29^ Trimmed fastq files were loaded into R version 4.0.0 (R Core Team, 2020) with the DADA2 package.^30^ Quality filtering was performed in DADA2 using the following criteria: trim = 0, maxEE = c(2,2), truncQ = 2, and rm.phix = TRUE. Filtered reads were run through the DADA2 amplicon sequence variant (ASV) assignment tool to denoise, cluster, and merge the reads. ASVs were assigned a taxon from the SILVA version 138.1 rRNA database^31^ using the Ribosomal Database Project (RDP) naïve Bayesian classifier.^32^ ASVs had to contain at least 0.05% of the total reads to remain in the dataset as well as be present in at least 1% of the samples, and were otherwise removed.

#### Statistical Analyses

Participants of both cohorts were combined: 225 participants with glaucoma were matched on age and sex with 1247 controls using propensity score matching, with the exact matching approach. In total, 165 (138 without glaucoma and 27 with glaucoma) non-matched participants were excluded from the analyses. Differences in characteristics between cases and controls were evaluated using chi-square tests and independent samples *t*-tests. We only analyzed taxonomical results at genus and species level (*N* = 233). Alpha- and beta-diversity indices were calculated based on these taxa. Kruskal-Wallis tests and permutational multivariate analysis of variance (PERMANOVA) test were utilized to determine if diversity was different between cases and controls. Relative abundances were calculated by dividing the raw count of a taxon by the total sum of all taxa, and multiplying by hundred. Taxa abundances (absolute counts +1) were then log transformed. We performed multivariable conditional logistic regression analyses for glaucoma and multivariable linear regression analyses for IOP and VCDR. The IOP and VCDR were log-transformed prior to the analyses. All analyses were adjusted for body mass index (BMI), use of medications, travelling, and technical covariates. The analyses for IOP and VCDR were additionally adjusted for age and sex. Statistical analyses were performed using RStudio (version 4.0.5; R Foundation for Statistical Computing, Vienna, Austria) with the add-on packages rbiom and Matchit. A *P* value < 0.05 or a Q-value < 0.20 (after adjusting for false discovery rate [FDR]; Benjamini-Hochberg)^33^ was considered statistically significant. An association was considered borderline significant when the *P* value was between 0.05 and 0.10.

### Replication Cohorts and Analyses

#### Study Populations and Available Data

The TwinsUK Adult Twin Registry is based at St. Thomas’ Hospital, London,^34^ and is the largest cohort of community-dwelling adult twins in the United Kingdom. Ethics approval for the TwinsUK study was given by the NRES Committee London-Westminster (REC Reference No.: EC04/015) and all participants provided informed consent. OCT and optic disc photographs were reviewed self-reported glaucoma cases. Glaucoma was definitive if the imaging supported a diagnosis (VCDR measured on OCT images >0.6, two clinicians judged the optic disc as suspicious or definite). IOP was measured with a non-contact air-puff tonometer,^35^ using the Ocular Response Analyser (ORA; Reichert, Buffalo, NY, USA) between 2006–2014 or the Visionix (Luneau Technology Operations, Pont-de-l'Arche, France) since 2014. The mean IOP was calculated from four readings (two from each eye). As glaucoma cases had a lower IOP than participants without glaucoma, it is likely they all received IOP-lowering treatment. Therefore, IOP for participants with glaucoma was imputed by increasing the measured value by 30%.^36^ Gut microbiota profiles from 16S rRNA gene sequencing of stool samples were available for approximately 3000 participants.^37^ We selected the twin with glaucoma or, when both twins were affected or unaffected, we randomly excluded one twin from each pair. The final analyses included 1574 unrelated participants.

Study of Health in Pomerania (SHIP) is a population-based project that assesses the prevalence and incidence of clinical diseases in West Pomerania, a region in the northeast of Germany.^38^ SHIP was approved by the Ethics Committee at the University Medicine Greifswald, Germany (approval number BB 39/08). Written informed consents were obtained from all participants. From the right eye, 45 degrees color fundus images were taken with a non-mydriatic camera (TRC-NW 200; Topcon Corporation, Tokyo, Japan). Pathological changes of the optic nerve were characterized.^39^ The VCDR was calculated by measuring the vertical diameter of the cup and the optic disc. A total of 2564 paired fecal samples from 1282 participants was available.^40^

#### Fecal Sample Collection and Microbiome Profiling

In the TwinsUK, profiles of gut microbiota composition were generated from fecal samples as previously described.^41^ Samples were sealed in ice packs by the participants and either sent via regular mail to the research department or handed over during the clinical visit. Samples were stored at −80°C and shipped frozen to Cornell University for DNA extraction and amplification of the V4 variable region of the 16S rRNA gene. DNA isolation was performed using the “MoBio PowerSoil htp DNA isolation kit” from an aliquot of approximately 100 mg of each sample.^41^ The QuantiT PicoGreen dsDNA Assay Kit was used to quantify the PCR amplicons, aliquots of which were combined for a final concentration of approximately 15 ng/µ. The resulting sequences were analyzed as ASVs following the DADA2 pipeline.^30^ DNA sequences were demultiplexed, and separate forward and reverse read files were generated for each sample using QIIME.^42^ Taxonomic assignment was via SILVA 1.3.2.^31^ Samples with less than 10,000 sequences or with only one viable read direction were removed.

In SHIP, 16S rRNA gene sequencing was performed as described elsewhere.^43^^,^^44^ Study participants collected fecal samples at their home and subsequently stored these in a tube containing stabilizing DNA buffer. The samples were then transported to the laboratory by the participants or courier. After DNA from fecal samples was isolated (PSP Spin Stool DNA Kit; Stratec Biomedical AG, Birkenfeld, Germany), it was stored at −20°C until analysis by 16S rRNA gene sequencing of the V1 to V2 region on a MiSeq platform (Illumina, San Diego, CA, USA). MiSeq FastQ files were created using CASAVA 1.8.2. The open-source software package DADA2 (version 1.10)^30^ was used for amplicon-data processing.^40^ All samples were normalized to 10,000 16S rRNA gene read counts for analysis. Samples with less than 10,000 sequences were removed.

#### Statistical Analyses

Differences in characteristics between cases and controls from the TwinsUK were evaluated using chi-square tests and independent samples *t*-tests. Alpha- and beta-diversity indices were calculated and the Kruskal-Wallis test and PERMANOVA test were utilized to determine if diversity was different between cases and controls. Taxa abundances were log+1 transformed. We performed multivariable logistic regression and linear regression analyses for glaucoma and IOP, respectively. IOP was log-transformed prior to the analyses. All analyses were adjusted for age, sex, BMI, use medications, and technical covariates. Subsequently, we conducted random-effects meta-analyses between the discovery cohort and TwinsUK.

In SHIP, taxa data were only available on genus level, and thus, in total, 60 genera were analyzed. Alpha-diversity indices were calculated based on these taxa. We performed multivariable linear regression analyses for VCDR. Taxa abundances were log+1 transformed preceding the analyses. All analyses were adjusted for age, sex, BMI, use medications, and the sequencing batch. Subsequently, we conducted random-effects meta-analyses between the discovery cohort and SHIP.

For the meta-analyses, we matched taxa of the discovery and replication cohorts on the assigned taxonomy name. Statistical analyses were performed using RStudio with the add-on package meta. A *P* value < 0.05 or a Q-value < 0.20 (after adjusting for FDR)^33^ was considered statistically significant. An association was considered borderline significant when the *P* value was between 0.05 and 0.10.

## Results

### Discovery Cohorts and Analyses

#### Baseline Characteristics

The baseline characteristics of participants from both discovery cohorts were comparable (Supplementary Table S1). Characteristics stratified on glaucoma status are presented in Table 1. In general, patients with glaucoma were older and had a lower BMI. As expected, their IOP was significantly higher and VCDR significantly larger.

**Table 1.** Characteristics of Age- and Sex-Matched Participants Included in the Discovery Cohort, Stratified on Glaucoma Status

	Glaucoma Present (*N* = 225)	Glaucoma Absent (*N* = 1247)	*P* Value
Age, y	70.2 (7.1)	63.6 (5.5)	<0.001^*^
Female sex, *N* (%)	122 (54.2)	711 (57.0)	0.48
BMI,^†^ kg/m^2^	25.1 (3.9)	27.4 (4.5)	<0.001^*^
Antibiotics,^‡^ *N* (%)	34 (15.1)	254 (20.4)	0.12
Probiotics,^§^ *N* (%)	22 (9.8)	126 (10.1)	>0.99
Winter production,^ǁ^ *N* (%)	163 (72.4)	393 (31.5)	<0.001^*^
Proton-pump inhibitors,^¶^ *N* (%)	59 (26.2)	245 (19.6)	0.03^*^
Lipid-lowering medications, *N* (%)	85 (37.8)	351 (28.1)	0.005^*^
Antidiabetics,^**^ *N* (%)	29 (12.9)	75 (6.0)	<0.001^*^
IOP,^††^ mm Hg	21.3 (7.7)	13.7 (2.9)	<0.001^*^
VCDR^‡‡^	0.8 (0.1)	0.5 (0.2)	<0.001^*^
Simpson's diversity indexi^§§^	0.89 (0.14)	0.92 (0.07)	0.001^*^
Inverse Simpson's diversity index^***^	16.3 (8.2)	17.6 (7.2)	0.03^*^
Shannon-Weiner diversity index	3.0 (0.8)	3.3 (0.5)	<0.001^*^

BMI, body mass index; IOP, intraocular pressure; *N*, number; VCDR, vertical cup-to-disc ratio.

Data are presented as mean (standard deviation), unless stated otherwise.

* *P* value < 0.05.

† Data available in 1469 participants (223 cases, 1246 controls).

‡ Data available in 1459 participants (217 cases, 1242 controls).

§ Data available in 1456 participants (216 cases, 1240 controls).

ǁ Data available in 1445 participants (214 cases, 1231 controls).

¶ Data available in 1469 participants (223 cases, 1246 controls).

** Data available in 1470 participants (224 cases, 1246 controls).

†† Data available in 1359 participants (185 cases, 1174 controls).

‡‡ Data available in 974 participants (8 cases, 966 controls).

§§ Data available in 1468 participants (223 cases, 1245 controls).

*** Data available in 1468 participants (223 cases, 1245 controls).

#### Intra- and Interindividual Diversity of the Gut Microbiomes

**Figure 1.**
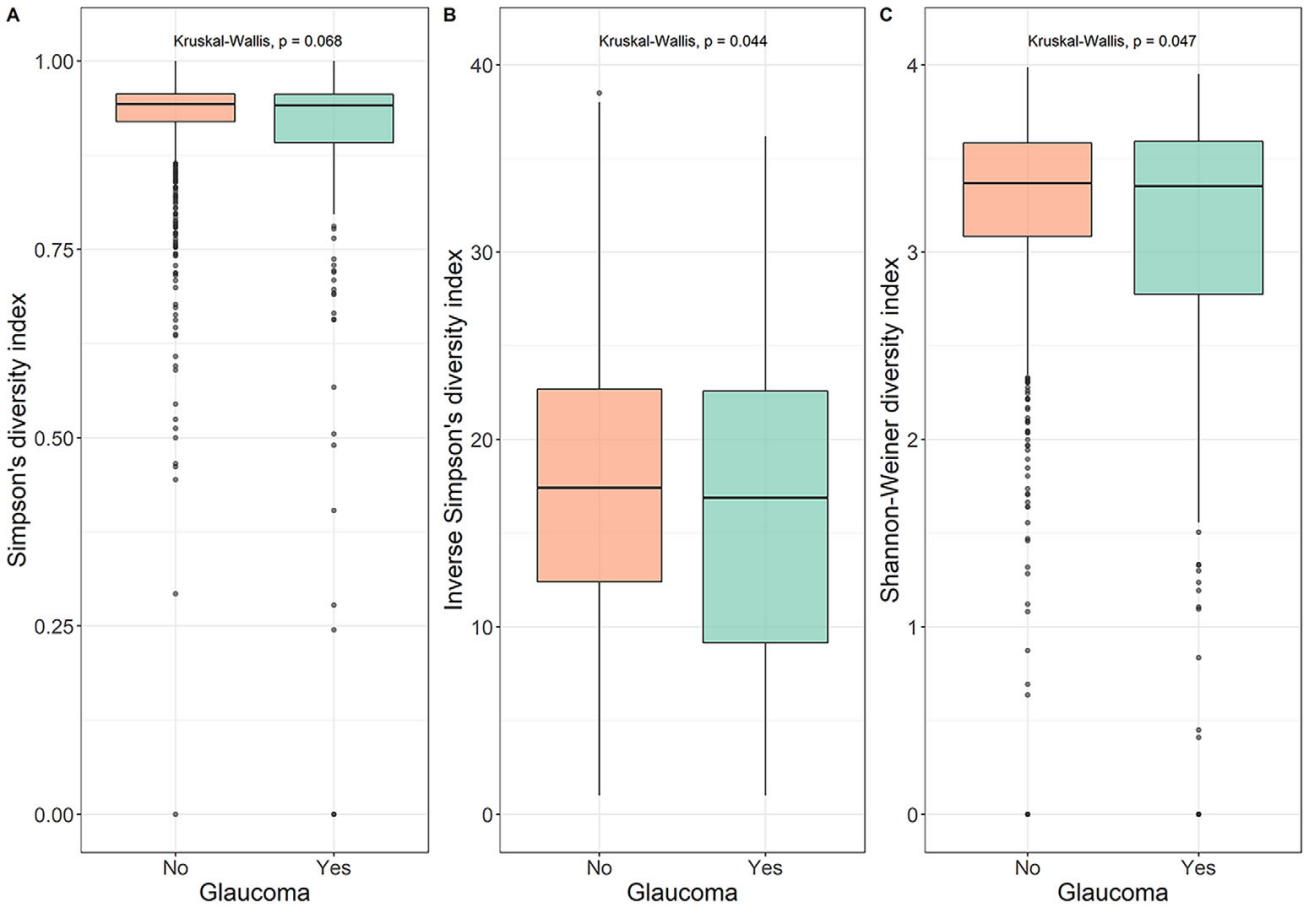
Alpha-diversity among participants without and with glaucoma of the discovery cohort. Diversity was not significant for Simpson's diversity index (**A**; *P* = 0.068, Kruskal-Wallis test), in contrast to inverse Simpson's diversity index (**B**; *P* = 0.044, Kruskal-Wallis test), and Shannon-Weiner diversity index (**C**; *P* = 0.047, Kruskal-Wallis test). *Boxes* indicate interquartile range (IQR) of 25th to 75th percentiles. The median value is shown as a *line within the box*. Whiskers extend to the most extreme value within 1.5 × IQR. Possible outliers are shown as *dots*.

**Figure 2.**
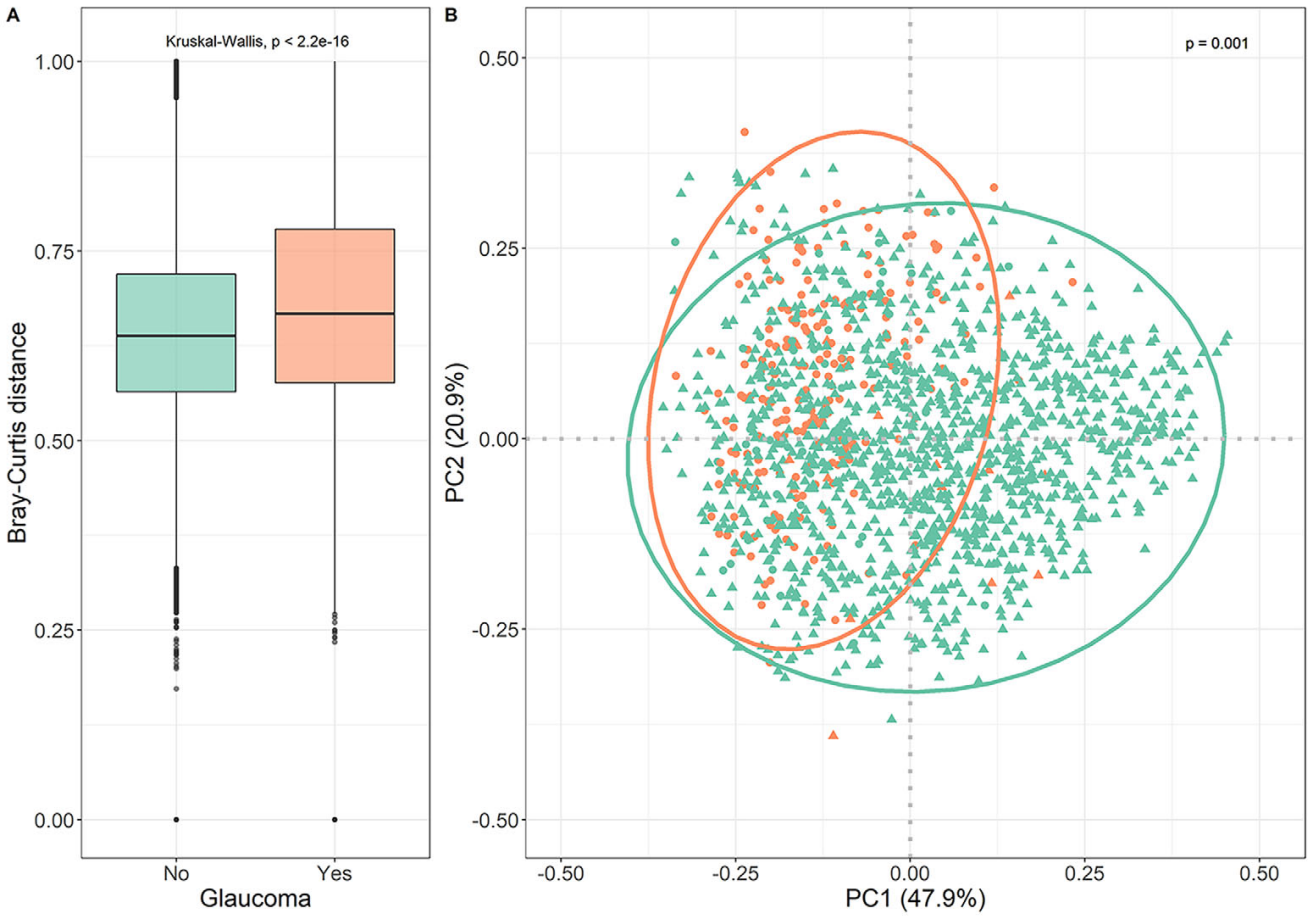
(**A**) Boxplot showing distance to the centroid and therefore the variance of Bray-Curtis distances within participants without and with glaucoma of the discovery cohort. The *P* value of the overall difference between groups obtained by Kruskal-Wallis test. (**B**) Principal Coordinate Analysis (PCoA) plots of beta diversity. Statistical significance between healthy participants (*green*) and patients with glaucoma (*orange*) using Bray-Curtis dissimilarity indices. Statistics were calculated using PERMANOVA with 999 permutations. Ellipses represent 95% confidence interval for each group. Dots include participants from Erasmus Glaucoma Cohort. *Triangles* include participants from Rotterdam Study.

**Figure 3.**
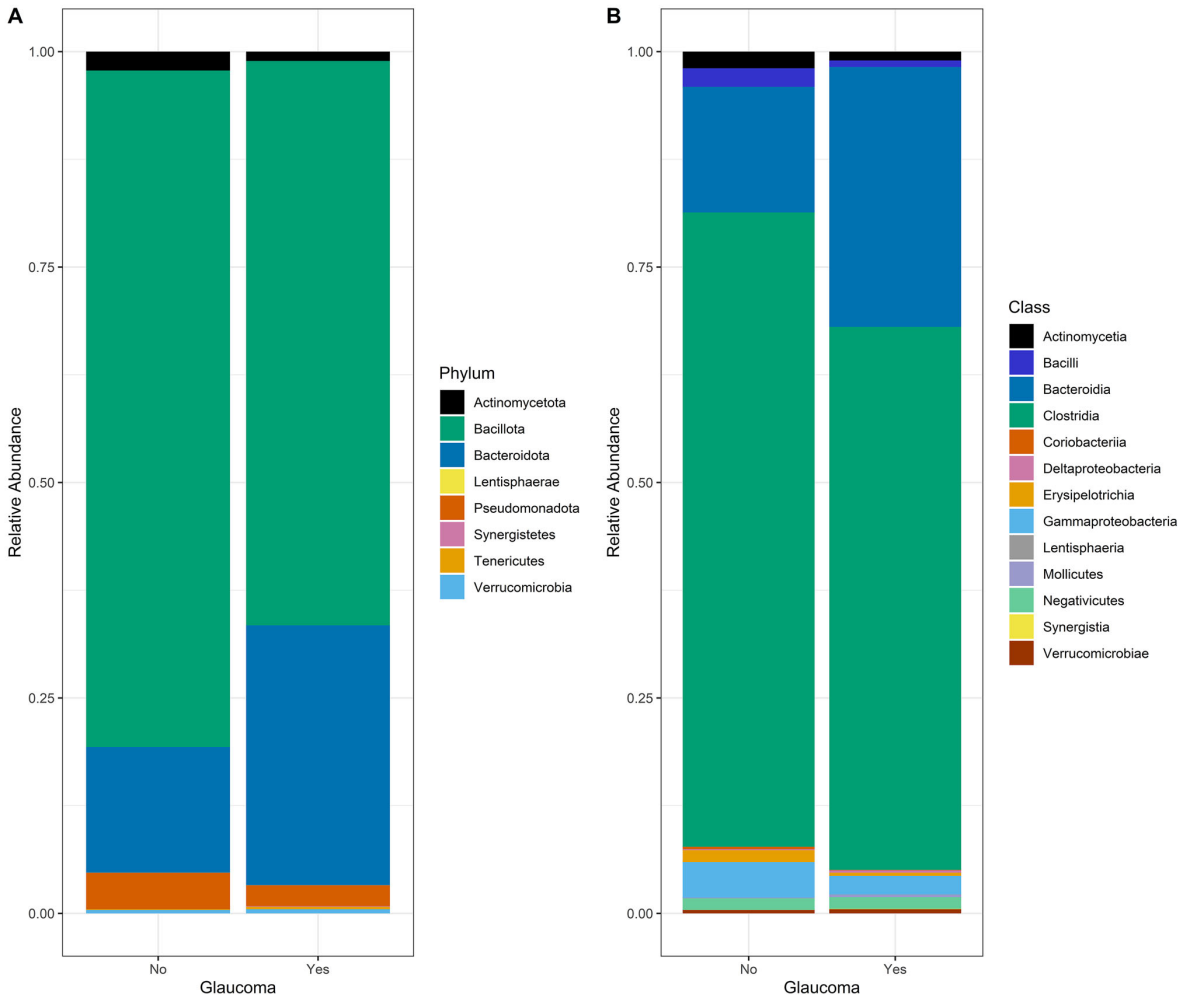
Overview of the most abundant phyla (**A**) and classes (**B**) present in age- and sex-matched participants of the discovery cohort: 225 patients with glaucoma matched with 1247 participants without glaucoma.

 Figure 1 displays different alpha-diversity indices in patients with glaucoma and healthy controls. Patients with glaucoma tended to have a somewhat lower alpha-diversity, including Simpson's diversity index (A; *P* value = 0.07), inverse Simpson's diversity index (B; *P* value = 0.04), and Shannon-Weiner diversity index (C; *P* value = 0.05). In the multivariate analysis, patients with glaucoma did not have a different microbial composition compared to healthy controls (Table 2). The Simpson's diversity index was associated with higher IOP. None of the indices were significantly associated with VCDR. Differences in the bacterial community compositions among all samples were assessed by Bray-Curtis dissimilarity (Fig. 2A) and principal component analysis (PCoA; Fig. 2B). Variance of the community composition was greater in patients with glaucoma than healthy controls (see Fig. 2A; *P* value < 2.2 × 10^−16^). The PCoA plot of beta-diversity evaluated by Bray-Curtis dissimilarity distances showed similarity/dissimilarity between the microbial composition from the two groups (*P* value = 0.001) However, due to the large sample number and interindividual variation, fecal microbiota for the two groups could not be clearly separated by PCoA (see Fig. 2B), even though there were significant differences in microbial community composition between the two groups according to the beta-diversity. Overall, participants with glaucoma had a lower relative abundance of the phylum Bacillota and a higher relative abundance of the phylum Bacteroidota (Fig. 3A). At class level (Fig. 3B), an increase in the relative abundance of Bacteroidia and a decrease in the relative abundance of Clostridia was observed in participants with glaucoma. Accounting for BMI, the use of probiotics or antidiabetic medications did not change these results (Supplementary Fig. S1).

**Table 2.** Associations of Alpha-Diversity With Glaucoma, Intraocular Pressure, and Vertical Cup-to-Disc Ratio

	Glaucoma		Intraocular Pressure		Vertical Cup-to-Disc Ratio	
	OR (95% CI)	*P* Value	Beta (95% CI)	*P* Value	Beta (95% CI)	*P* Value
Simpson's diversity index	2.38 (0.26 to 21.77)	0.44	0.18990 (0.00256 to 0.37716)	0.05^*^	−0.00579 (−0.19164 to 0.18006)	0.95
Inverse Simpson's diversity index	1.01 (0.98 to 1.05)	0.43	0.00099 (−0.00072 to 0.00270)	0.26	−0.00041 (−0.00185 to 0.00104)	0.58
Shannon-Weiner diversity index	1.14 (0.80 to 1.64)	0.47	0.02069 (−0.00717 to 0.04855)	0.15	−0.00179 (−0.02782 to 0.02424)	0.89

CI, confidence interval; OR, odds ratio.

All glaucoma analyses were adjusted for body mass index, use of antibiotics, probiotics, proton-pump inhibitors, lipid-lowering medications and antidiabetics, travelling, and technical covariates (time sample has spent in the mail, the season in which the sample was produced, the number of reads, the DNA isolation batch and the sequencing batch). Intraocular pressure and vertical cup-to-disc ratio analyses were additionally adjusted for age and sex.

* *P* value < 0.05.

#### Associations of Taxa With Glaucoma, IOP, and VCDR

Nine taxa showed a significantly different abundance in people with glaucoma compared to healthy controls; 16 taxa barely missed statistical significance (Supplementary Table S2). For IOP, 23 taxa were significantly associated and 23 taxa borderline significantly associated (see Supplementary Table S2). Interestingly, two taxa that were associated, and were more abundant in healthy controls also showed an association with lower IOP: *Anaerosporobacter* (Beta, 95% confidence interval [CI], Beta = −0.01031, 95% CI = −0.01847 to −0.00215) and *Butyrivibrio* (Beta = −0.01015, 95% CI = −0.02010 to −0.00021). For VCDR, we identified 15 taxa that were significantly and 11 taxa that were borderline significantly associated (see Supplementary Table S2). *Caproiciproducens* and *Clostridium sensu stricto 1* were associated both with lower IOP (Beta = −0.01590, 95% CI = −0.02743 to −0.00438 and Beta = −0.00471, 95% CI = −0.00958 to 0.00015) and a smaller VCDR (Beta = −0.00615, 95% CI = −0.01277 to 0.00047 and Beta = −0.00327, 95% CI = −0.00602 to −0.00051). None of the taxa remained significantly associated (Q-value < 0.20) with glaucoma, IOP, or VCDR after adjusting for FDR.

### Replication Cohorts and Analyses

#### Baseline Characteristics

The TwinsUK dataset consisted of 1574 participants (including 32 participants with glaucoma and 1542 unrelated, unmatched participants without glaucoma). Characteristics stratified on glaucoma status are presented in Supplementary Table S3. The SHIP dataset consisted of a total of 2546 participants (Supplementary Table S4).

#### Intra- and Interindividual Diversity of the Gut Microbiomes

In TwinsUK, participants with glaucoma did not have a different microbial composition compared to healthy controls, as neither the Simpson's diversity index nor the Shannon-Weiner diversity index were significantly associated with glaucoma status. Moreover, both indices were not associated with IOP. When meta-analyzing results of the discovery cohort and results of TwinsUK, we confirmed these null-associations (Table 3). However, there was a significant positive association between the Simpson's diversity index and IOP (Beta = 0.19648, 95% CI = 0.03288–0.36009). In SHIP, neither the Inverse Simpson's diversity index nor the Shannon-Weiner diversity index were significantly associated with VCDR. When meta-analyzing results of the discovery cohort and results of SHIP, we confirmed these null-associations (see Table 3). When assessing differences in the bacterial community compositions among the TwinsUK samples, utilizing Bray-Curtis dissimilarity (Supplementary Fig. S2A) and PCoA (Supplementary Fig. S2B), we observed a greater variance of the community composition in patients with glaucoma than healthy controls (see Supplementary Fig. S2A; *P* value = 0.004). The PCoA plot of beta-diversity evaluated by Bray-Curtis dissimilarity distances showed no similarity/dissimilarity between the microbial composition from the two groups (*P* value = 0.44).

**Table 3.** Meta-Analyzed Associations (Results of Discovery Cohort With Results of TwinsUK and the Study of Health in Pomerania [SHIP]) of Alpha-Diversity With Glaucoma, Intraocular Pressure, and Vertical Cup-to-Disc Ratio

	Glaucoma		Intraocular Pressure		Vertical Cup-to-Disc Ratio	
	OR (95% CI)	*P* Value	Beta (95% CI)	*P* Value	Beta (95% CI)	*P* value
Simpson's diversity index	1.61 (0.17 to 15.68)	0.68	0.19648 (0.03288 to 0.36009)	0.02^*^	–	–
Inverse Simpson's diversity index	–	–	–	–	−0.00011 (−0.00032 to 0.00011)	0.34
Shannon-Weiner diversity index	1.12 (0.80 to 1.56)	0.52	0.02060 (−0.00038 to 0.04157)	0.05	−0.00423 (−0.01422 to 0.00577)	0.41

CI, confidence interval; OR, odds ratio.

Random-effects meta-analyses assessing the association between alpha diversity and glaucoma, intraocular pressure, and vertical cup-to-disc ratio. The analyses from the discovery cohort were adjusted for body mass index (BMI), use of antibiotics, probiotics, proton-pump inhibitors, lipid-lowering medications and antidiabetics, travelling, and technical covariates (time sample has spent in the mail, the season in which the sample was produced, the number of reads, the DNA isolation batch and the sequencing batch). The analyses for intraocular pressure and vertical cup-to-disc ratio were additionally adjusted for age and sex. In TwinsUK, all analyses were adjusted for aforementioned covariates. Data on use of probiotics and travelling was not available. In SHIP, all analyses were adjusted for age, sex, BMI, use of proton-pump inhibitors, lipid-lowering medications and antidiabetics, and the sequencing batch.

* *P* value < 0.05.

#### Associations of Taxa With Glaucoma, IOP, and VCDR

**Figure 4.**
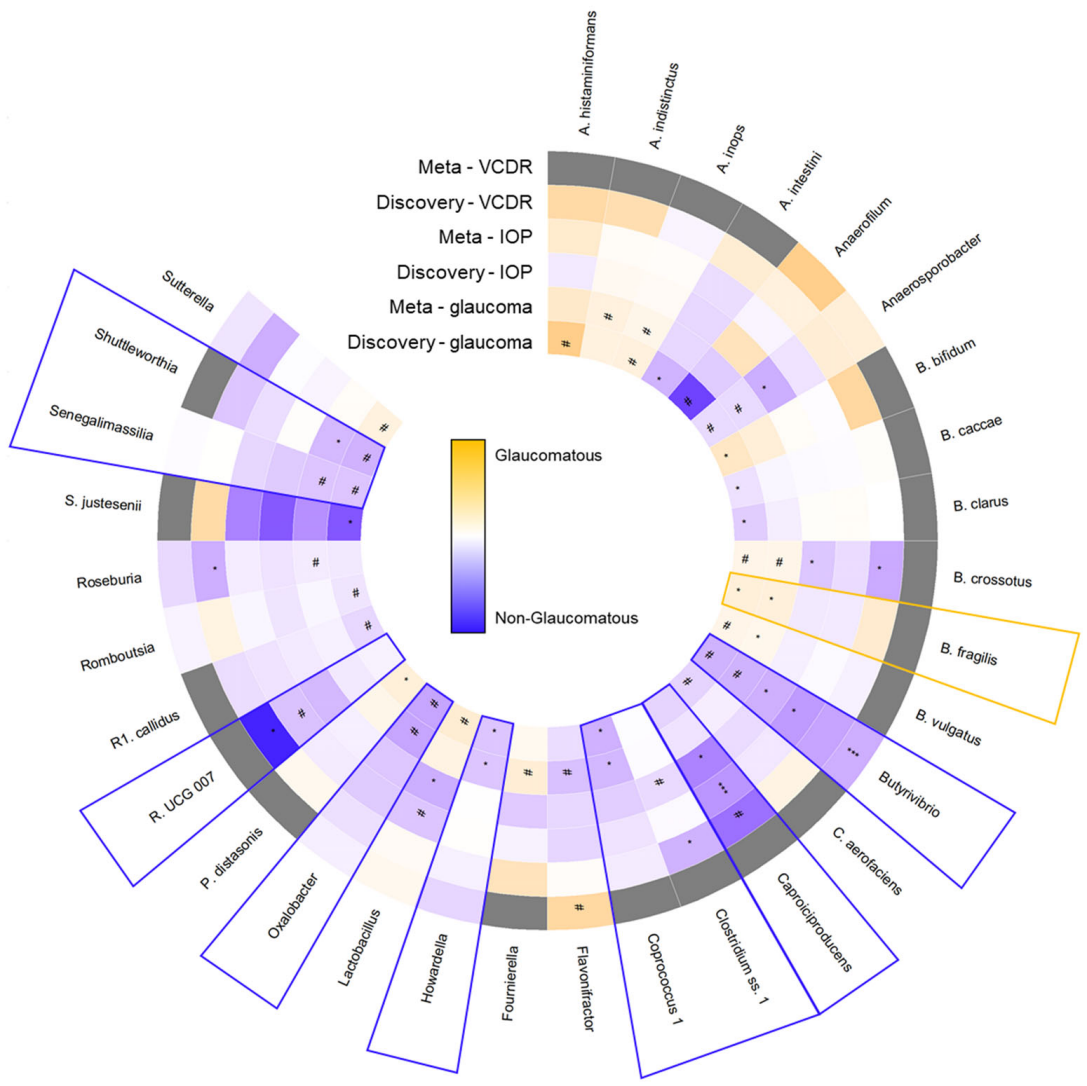
Individual taxa, on genus and species level, associated with glaucoma (in either the discovery cohort or meta-analyses), intraocular pressure (IOP; in both the discovery cohort and meta-analyses), and vertical cup-to-disc ratio (VCDR; in both the discovery cohort and meta-analyses). Results are displayed on a non-glaucomatous (*blue* = lower odds of glaucoma [range = betas −1.00 to 0.00], lower IOP [range = betas −0.03 to 0.00], and smaller VCDR [range = betas −0.01 to 0.00]) to glaucomatous (*orange* = higher odds of glaucoma [range = betas 0.00 to 1.00], higher IOP [range = betas 0.00 to 0.03], and larger VCDR [range = betas 0.00 to 0.01]) scale. Grey color indicates that the analysis was not performed due to missing data. *** Q-value < 0.20 (false discovery rate adjusted); * *P* value < 0.05; # 0.05 < *P* value < 0.10.

In the TwinsUK dataset, 226 (97.0%) of the 233 taxa from the discovery cohort were present. For glaucoma, none of the taxa identified in the discovery cohort replicated within the TwinsUK (Supplementary Table S5). Therefore, we meta-analyzed the results on glaucoma of our discovery cohort (see Supplementary Table S2) with the TwinsUK (see Supplementary Table S5). Taxa that had a significantly different abundance in people with glaucoma compared to healthy controls in either the discovery analyses or the meta-analyses are displayed in Figure 4 (see the full results presented in Supplementary Table S6). Three of the previously associated taxa retained their significance: *Bacteroides fragilis* (odds ratio [OR] = 1.17, 95% CI = 1.02–1.35), *Coprococcus 1* (OR = 0.74, 95% CI = 0.58–0.94), and *Howardella* (OR = 0.79, 95% CI = 0.62–1.00). Additionally, two taxa that previously showed a borderline significant association gained *P* values below the significance threshold: *Bacteroides vulgatus* (OR = 1.09, 95% CI = 1.00–1.18) and *Shuttleworthia* (OR = 0.74, 95% CI = 0.57–0.97).

In addition, six taxa that were previously found to differ (with borderline significance) in abundance between cases and controls, retained borderline significant associations in the meta-analyses. Moreover, the meta-analyses revealed that the abundance of four additional taxa was different between cases and controls.

For IOP, four of the 44 taxa identified in the discovery cohort replicated within the TwinsUK (see Supplementary Table S5): *Butyrivibrio* (Beta = −0.02195, 95% CI = −0.04151 to –0.00239), *Caproiciproducens* (Beta = −0.01099, 95% CI = −0.02337 to 0.00138), *Ruminococcaceae UCG 008* (Beta = −0.00976, 95% CI = −0.02099 to 0.00146), and *UBA1819* (Beta = −0.01131, 95% CI = −0.02221 to –0.00040). We meta-analyzed the results on IOP and VCDR of our discovery cohort (see Supplementary Table S2) with the TwinsUK (see Supplementary Table S5). Taxa that were associated with both IOP and VCDR (see below) in either the discovery or the meta-analyses (with estimates in the same direction) are displayed in Figure 4 (the full results are presented in Supplementary Table S6). Seven of the previously associated taxa retained their statistical significance (*Caproiciproducens* gained statistical significance after FDR correction; Q-value = 0.17); four taxa that previously showed borderline associations with IOP gained *P* values below the significance threshold; eight previously borderline associated taxa retained borderline significant associations; and one additional taxon was associated with IOP. Of these, *Butyrivibrio* was less abundant in patients with glaucoma (OR = 0.72, 95% CI = 0.51–1.01) and was associated with lower IOP (Beta = −0.01292, 95% CI = −0.02272 to −0.00313) when meta-analyzing the results from the discovery cohort and TwinsUK.

In the SHIP dataset, 60 (25.8%) of the 233 taxa from the discovery cohort were present, as data were available only on genus level. For VCDR, none of the taxa identified in the discovery cohort replicated within SHIP (Supplementary Table S7). Therefore, we meta-analyzed the results on VCDR of the discovery cohort (see Supplementary Table S2) with SHIP (see Supplementary Table S7). Taxa that were associated with both IOP (see above) and VCDR in either the discovery or meta-analyses (with estimates in the same direction) are displayed in Figure 4 (see the full results presented in Supplementary Table S8). Only one of the previously associated taxa retained its significance: *Streptococcus* (Beta = 0.00224, 95% CI = 0.00031–0.00418), but this taxon was not associated with IOP. Moreover, the meta-analyses revealed four additional taxa to be significantly or borderline significantly associated with VCDR. *Butyrivibrio* was significantly associated with a smaller VCDR (Beta = −0.00345, 95% CI = −0.00565 to –0.00126; Q-value = 0.12). This taxon was also less abundant in patients with glaucoma and associated with lower IOP.

None of the taxa remained significantly associated (Q-value < 0.20) with glaucoma, IOP, or VCDR after adjusting for FDR, unless stated otherwise.

## Discussion

We observed and replicated consistent associations between the gut microbiome and glaucoma in multiple independent cohort studies. We observed that several butyrate-producing taxa were less abundant in patients with glaucoma compared to healthy controls. Similar protective associations were seen with IOP and VCDR.

In general, alpha-diversity tended to be lower in patients with glaucoma compared to healthy controls. Moreover, although the two groups could not be clearly separated by PCoA, there were significant differences in microbial community composition between the two groups according to the beta-diversity in the discovery cohort, but not in the replication cohort (TwinsUK). Gong et al. did not observe any significant differences in alpha (Shannon-Weiner diversity index) or beta-diversity (unweighted UniFrac) between glaucoma cases and controls.^45^ However, they analyzed gut microbiome from primary angle-closure glaucoma, whereas we included mainly primary open-angle glaucoma cases. The visual representation of the gut microbial composition of participants with and without glaucoma showed that patients with glaucoma had a lower Bacillota/Bacteroidota ratio than participants without glaucoma. Contradicting, Zhang et al. observed an increased Bacillota/Bacteroidota ratio in the intestinal flora of animals with glaucoma.^46^

Although only very few associations on genus and species level survived multiple testing correction, we would like to highlight a group of taxa, namely those producing butyrate, whose abundance was lower in patients with glaucoma compared to healthy controls. Butyrate is a short-chain fatty acid (SCFA) produced by bacterial fermentation of fiber in the colon. Butyrate can exert direct immuno-modulatory effects, including the suppression of nuclear factor kappa B (NF-κB) activation.^47^^–^^49^ Chen et al. identified NF-κB subunit 1 (NFKB1) as one of the hub genes in glaucoma that are gut microbiome-related.^50^ These hub genes may affect glaucoma progression through several metabolites, including butyrate. Schulthess et al. demonstrated that butyrate can imprint potent antimicrobial activity during macrophage differentiation.^51^ Macrophages maintain gut homeostasis by eliminating invasive pathogens and regulating inflammatory responses. Nevertheless, gut homeostasis is also influenced by other external and internal factors, including dietary habits,^52^ physical exercise,^53^ and use of antibiotics.^54^ A low-fiber diet, which leads to a lower production of SCFAs including butyrate, can lead to the disruption of the mucous layer and the tight junctions in the gut. Subsequently, a shift to a pro-inflammatory microbiome occurs, producing higher levels of TNF-alfa, IL-6, and IL-1.^55^ These detrimental effects may be reversed by an antioxidant diet which can increase fiber-fermenting and butyrate-producing bacteria, increasing the tightness of the intestinal barrier.^56^^,^^57^ Research has shown that microbiota-driven gut leakiness is involved in the development of other neurodegenerative disorders, like Parkinson's disease and Alzheimer's disease.^58^ Moreover, in elderly people, a decrease in the level of SCFAs from carbohydrate fermentation is observed, whereas metabolites from protein fermentation (branched fatty acids, ammonia, and phenols) are increased. This indicates a shift from saccharolytic fermentation to unfavorable proteolytic activities.^59^^,^^60^ This shift continues to progress as elderly people age,^61^ and occurs more rapidly with the use of antibiotics or low-fiber diets.^60^^,^^62^^,^^63^ These findings not only suggest that a “leaky gut” may also be involved in glaucoma (a disease associated with older age), but also highlight the importance of a healthy diet for patients with or at risk of glaucoma. Particularly because disruption of gut barrier function has shown to enable the migration of microbial products and immune cells into the eye.^64^

In addition to the inhibition of NF-κB, the anti-inflammatory activity of butyrate may be established through the inhibition of interferon-γ production and/or signaling,^65^^,^^66^ and the upregulation of peroxisome proliferator-activated receptor-γ (PPARγ).^67^^–^^69^ Pioglitazone, an agonist of PPARγ used in the treatment of type 2 diabetes mellitus, has been shown to significantly protect RGCs and prevent axonal degeneration in the glaucomatous retina of mice.^70^ Furthermore, treatment with pioglitazone preserved and partially reversed vision loss in spite of continuously elevated IOP. Similarly, two other PPARγ ligands protected transformed rat RGC against glutamate cytotoxicity.^71^ The neuroprotective effects of these two compounds appeared to be PPARγ-independent, suggesting that PPARγ agonists may also provide a valuable antioxidant benefit.

Research focused on butyrate itself has shown that butyrate is able to lower IOP in normotensive but not in hypertensive rat eyes.^72^ Similarly, in patients with suspected steroid-induced glaucoma, clobetasone butyrate eye drops limited the steroid-induced increase in IOP, whereas betamethasone phosphate (i.e. without butyrate) significantly raised the IOP.^73^ Sodium phenylbutyrate (PBA) is a derivative of the SCFA butyrate. Topical administration of PBA has shown to reduce glaucomatous phenotypes in a mouse model of myocilin-associated glaucoma.^74^ PBA rescues cells from endoplasmic reticulum stress and apoptosis.^75^ Likewise, Maddineni et al. demonstrated that PBA reduces ocular hypertension by degrading extracellular matrix deposition of the trabecular meshwork.^76^ There are very limited studies that have investigated the association between the gut microbiome and glaucoma, and their sample sizes have been small.^77^^,^^78^ The strengths of this study include the extensive phenotyping, that is, the availability of not only glaucoma diagnosis but also continuous glaucoma-associated parameters, such as IOP and VCDR. In the discovery cohort, only visual field testing was included in the definition of glaucoma. Therefore, by analyzing the IOP and VCDR we were able to confirm the association between the gut microbiome and glaucoma in an independent manner.

We were limited by the use of cross-sectional data, thus, the observed microbial composition does not per definition reflect long term microbial composition.^79^ Moreover, the design limits causal inference. Future investigations using longitudinal cohort studies, assessing microbial composition at multiple time points, are warranted to elucidate dynamic microbial changes. These studies could utilize predictive methodologies to potentially predict outcomes within microbial communities over time. Although we adjusted for a large number of covariates in our analyses, it is possible that other potentially important confounders were not included. Because dietary intake is associated with both the microbiome^80^^–^^83^ and glaucoma,^84^^–^^86^ it is possible that disregarding this variable has distorted our findings. Moreover, residual confounding remains a potential issue. When generating the Bacillota/Bacteroidota ratio plot, we took into account confounding by, for example, BMI and use of probiotics or antidiabetic medications. However, after additional matching on BMI or after removing any participants using probiotics or antidiabetic medications, patients with glaucoma still had a lower Bacillota/Bacteroidota ratio than healthy controls (see Supplementary Fig. S1). Although this does not directly imply that these confounders do not have any residual effect on the association between the gut microbiome and glaucoma on genus and species level, the consistency in the composition does reinforce the robustness of our findings. Another limitation is the use of different methods for the fecal sample collection. As the largest proportion of patients with glaucoma was derived from the EGC and the participants without glaucoma were mainly selected from RS, this could have introduced differential-misclassification. We observed a significant difference in beta-diversity between cases and controls of the discovery cohort, but not in TwinsUK, which suggests this was potentially driven by a batch effect. This batch effect may obscure any real difference in beta-diversity between people with and without glaucoma. In our multivariate analysis, we tried to mitigate this by adjusting for technical covariates. In addition, we cannot completely rule out that glaucoma was absent in the healthy participants (*N* = 67) from EGC, as these participants were not seen by an ophthalmologist. Last, only very few associations remained statistically significant after adjusting for FDR. As this study has an exploratory character, and requires replication and validation, a strict adjustment for multiple comparisons is less critical, and focusing on effect sizes and confidence intervals (CIs) rather than tests of significance may be more suitable.^87^

In conclusion, our data suggest that butyrate-producing taxa may play a role in the pathophysiology of glaucoma. Prospective studies with multiple follow-up visits are needed to account for changing microbial compositions and to make causal inferences. Future research should also aim to assess whether butyrate-producing taxa are potential mediators in the relation between dietary intake and glaucoma. If so, dietary recommendations or supplements targeting these taxa specifically, may become available for the prevention and treatment of glaucoma.

## Supplementary Material

Supplement 1

Supplement 2

Supplement 3

Supplement 4

Supplement 5

Supplement 6
