# Supplementary material for: Glaucoma Patients Have a Lower Abundance of Butyrate-Producing Taxa in the Gut
Source: Invest Ophthalmol Vis Sci. 2024 Feb 5;65(2):7. doi: 10.1167/iovs.65.2.7 (PMC10851784; doi:10.1167/iovs.65.2.7)
Supplement: Supplement 1 [file iovs-65-2-7_s001.pdf]

Supplementary Table S1. Characteristics of age- and sex-matched participants with and without glaucoma included in the discovery analyses, stratified on cohort

	**Glaucoma present**			**Glaucoma absent**		
	RS (N=15)	EGC (N=210)	P-value	RS (N=1180)	EGC (N=67)	P-value
**Age, years**	66.5 (5.2)	70.5 (7.1)	0.01*	63.1 (5.2)	71.3 (5.4)	<0.001*
**Female sex, N (%)**	7 (46.7)	115 (54.8)	0.73	679 (57.5)	32 (47.8)	0.15
**BMI, kg/m^2**	26.4 (4.0)	25.0 (3.9)	0.22	27.5 (4.5)	26.2 (4.7)	0.04*
**Antibiotics, N (%)**	4 (26.7)	30 (14.3)	0.26	249 (21.1)	5 (7.5)	0.01*
**Probiotics, N (%)**	2 (13.3)	20 (9.5)	0.65	118 (10.0)	8 (11.9)	0.77
**Winter production, N (%)**	7 (46.7)	156 (74.3)	<0.001*	336 (28.5)	57 (85.1)	<0.001*
**Proton-pump inhibitors, N (%)**	3 (20.0)	56 (26.7)	0.76	233 (19.7)	12 (17.9)	0.88
**Lipid-lowering medications, N (%)**	4 (26.7)	81 (38.6)	0.42	334 (28.3)	17 (25.4)	0.70
**Antidiabetics, N (%)**	2 (13.3)	27 (12.9)	>0.99	72 (6.1)	3 (4.5)	0.79
**IOP, mmHg**	17.2 (4.7)	21.7 (7.8)	0.003*	13.7 (2.9)	-	-
**VCDR**	0.8 (0.1)	-	-	0.5 (0.2)	-	-
**Simpson's diversity index**	1.0 (0.0)	0.9 (0.1)	<0.001*	1.0 (0.0)	0.9 (0.2)	0.01*
**Inverse Simpson's diversity index**	71.5 (11.7)	54.4 (26.9)	<0.001*	66.7 (16.5)	54.2 (29.5)	0.001*
**Shannon-Weiner diversity index**	4.3 (0.2)	3.7 (1.0)	<0.001*	4.2 (0.3)	3.6 (1.3)	<0.001*

*Data are presented as mean (standard deviation), unless stated otherwise. * P-value<0.05. Abbreviations: RS = Rotterdam Study; EGC = Erasmus Glaucoma Cohort; N = number; BMI = body mass index; IOP = intraocular pressure; VCDR = vertical cup-to-disc ratio.*

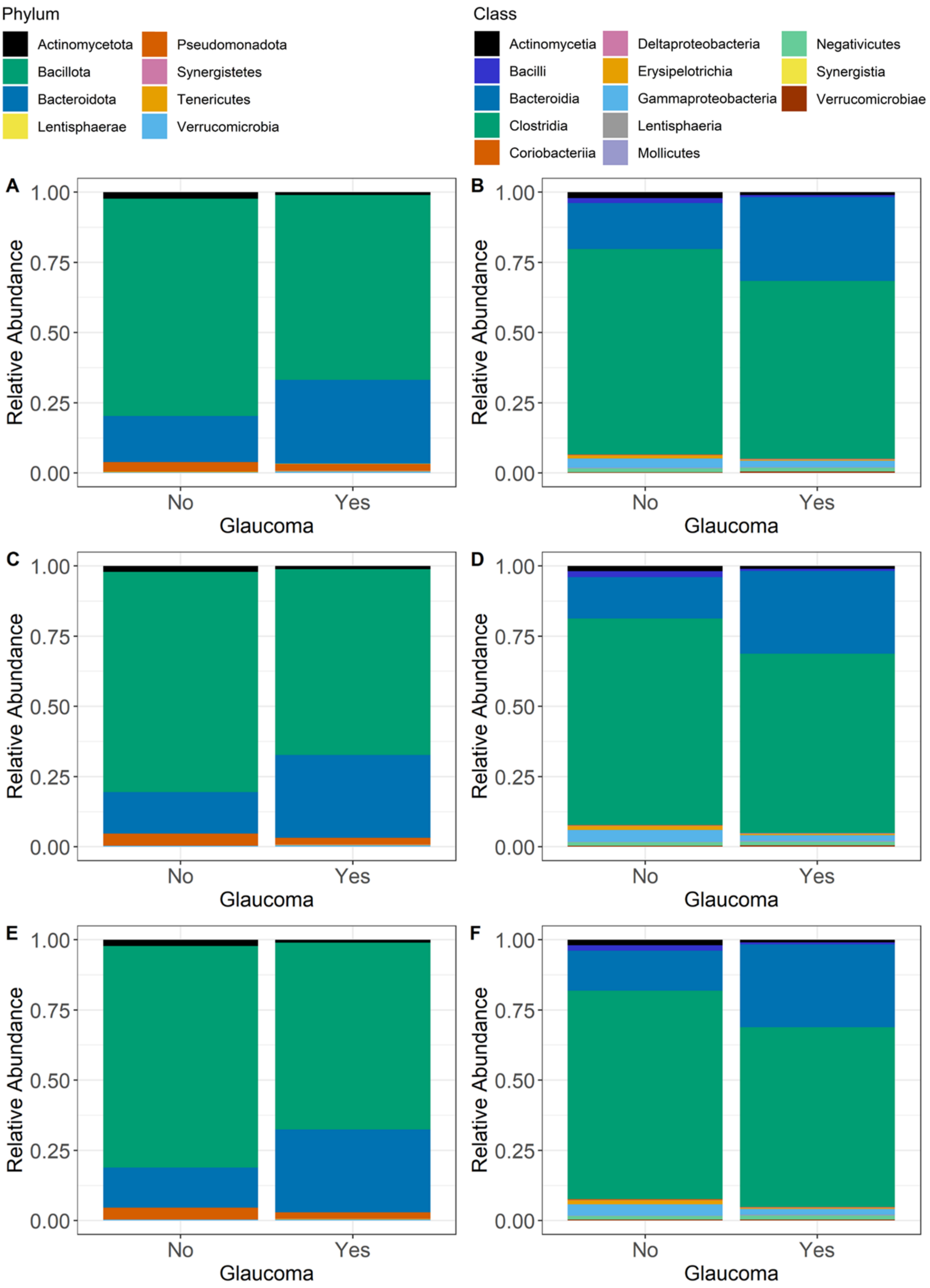
Phylum
Actinomycetota
Bacillota
Bacteroidota
Lentisphaerae
Pseudomonadota
Synergistetes
Tenericutes
Verrucomicrobia
Class
Actinomycetia
Bacilli
Bacteroidia
Clostridia
Coriobacteriia
Deltaproteobacteria
Erysipelotrichia
Gammaproteobacteria
Lentisphaeria
Mollicutes
Negativicutes
Synergistia
Verrucomicrobiae
A
B
C
D
E
F
Relative Abundance
1.00
0.75
0.50
0.25
0.00
No
Yes
Glaucoma

Supplementary Figure S1. Relative abundance of the different phyla (A) and classes (B) in age, sex, and body mass index-matched participants of the discovery cohort: 223 glaucoma patients matched with 223 healthy individuals. Relative abundance of the different phyla (C) and classes (D) in age and sex-matched participants of the discovery cohort after excluding probiotics users: 194 glaucoma patients matched with 1093 healthy individuals. Relative abundance of the different phyla (E) and classes (F) in age and sex-matched participants of the discovery cohort after excluding antidiabetic medication users: 195 glaucoma patients matched with 1156 healthy individuals.

Supplementary Table S3. Characteristics of participants included in the replication analysis (TwinsUK), stratified on glaucoma status

	Glaucoma present (N = 32)	Glaucoma absent (N = 1542)	P-value
**Age, years**	62.4 (10.4)	59.6 (12.4)	0.07
**Female sex, N (%)**	30 (93.8)	1363 (88.4)	0.17
**IOP, mmHg**	13.9 (10.4)	14.6 (12.4)	0.31
**BMI, kg/m^2**	25.0 (3.9)	26.1 (4.9)	0.06
**Antibiotics, N (%)**	0 (0.0)	50 (3.2)	-
**Winter production, N (%)**	8 (25.0)	438 (28.4)	0.34
**Proton-pump inhibitors, N (%)**	9 (28.1)	300 (19.5)	0.11
**Lipid-lowering medications, N (%)**	10 (31.3)	343 (22.2)	0.11
**Antidiabetic medications, N (%)**	3 (9.4)	53 (3.4)	0.04*
**Simpson's diversity index**	0.93 (0.07)	0.94 (0.04)	0.33
**Shannon-Weiner diversity index**	3.8 (0.6)	3.8 (0.5)	0.38

*Data are presented as mean (standard deviation), unless stated otherwise. * P<0.05. Abbreviations: N = number; IOP = intraocular pressure; BMI = body mass index.*

Supplementary Table S4. Characteristics of participants included in the replication analysis (the Study of Health in Pomerania [SHIP])

	**Total population (N = 2546)**
**Age, years**	49.5 (14.3)
**Female sex, N (%)**	1292 (50.8)
**BMI, kg/m^2**	27.8 (5.1)
**VCDR**	0.4 (0.1)
**Antibiotics, N (%)**	0 (0.0)
**Proton-pump inhibitors, N (%)**	194 (7.6)
**Lipid-lowering medications, N (%)**	294 (11.6)
**Antidiabetics, N (%)**	150 (5.9)
**Inverse Simpson's diversity index**	42.7 (19.1)
**Shannon-Weiner diversity index**	4.4 (0.4)

Data are presented as mean (standard deviation), unless stated otherwise. Abbreviations: N = number; BMI = body mass index; VCDR = vertical cup-to-disc ratio.

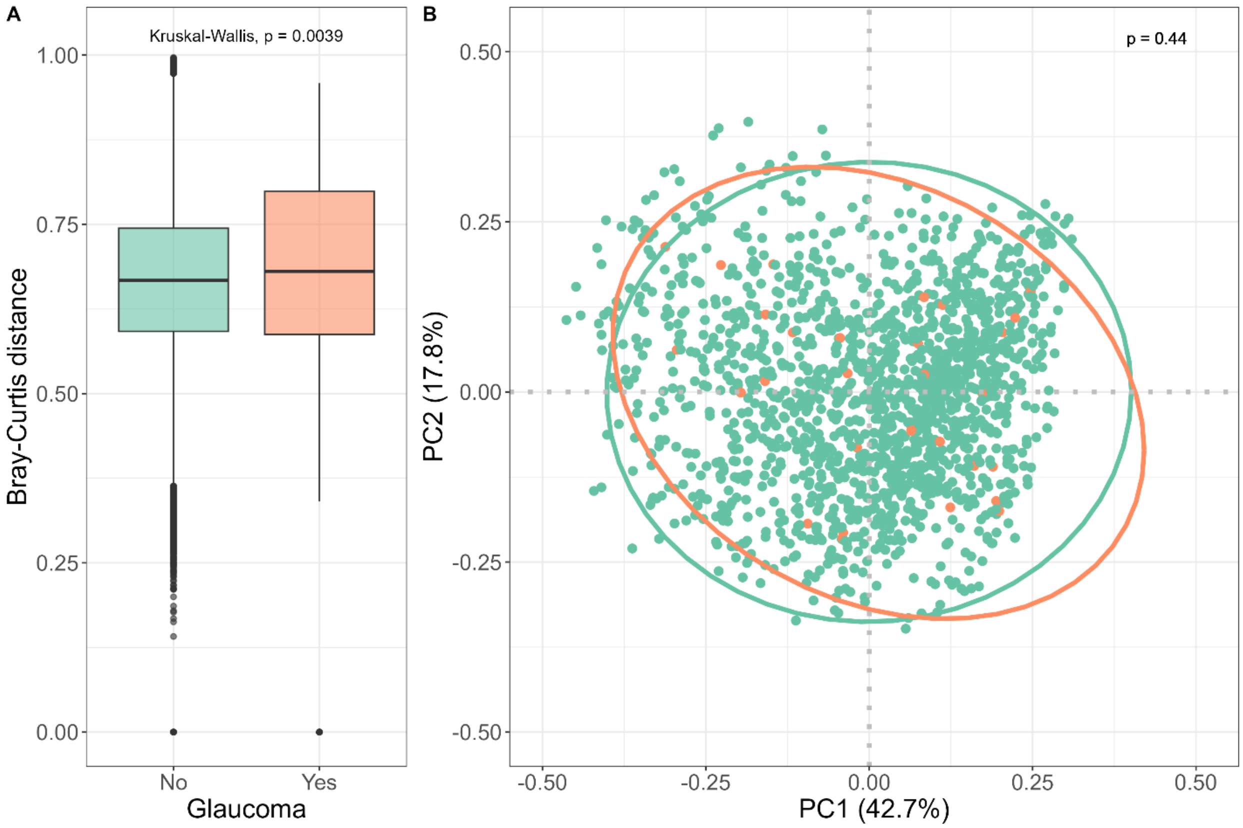

Supplementary Figure S2. A) Boxplot showing distance to the centroid and therefore the variance of Bray-Curtis distances within participants without and with glaucoma from TwinsUK. The P-value of the overall difference between groups obtained by Kruskal-Wallis test. B) Principal Coordinate Analysis (PCoA) plots of beta diversity of participants from TwinsUK. Statistical significance between healthy participants (green) and glaucoma patients (orange) using Bray-Curtis dissimilarity indices. Statistics were calculated using PERMANOVA with 999 permutations. Ellipses represent 95% confidence interval for each group.
